# In Response to Gürdoğan and Akkuş

**DOI:** 10.4274/balkanmedj.galenos.2019.2019.11.148-response

**Published:** 2020-02-28

**Authors:** Hasan Ali Barman

**Affiliations:** 1 Clinic of Cardiology, İstanbul Okmeydanı Training and Research Hospital, İstanbul, Turkey

To the Editor,

We would like to thank the authors for their interest in our paper (1) and their valuable comments. In the authors’ letter to the editor, they mentioned potential concerns regarding the diagnosis of Fabry disease in female patients.

The main aim of our study (1) was to screen adult patients with hypertrophic cardiomyopathy without left ventricular outflow tract. In the diagnosis of Fabry disease, plasma α-galactosidase A activity and plasma and urine glycosphingolipid measurements as well as molecular genetic analyses of the GLA gene are used (2). Fabry disease is an X-linked disorder. According to the Lyon hypothesis, in case of X chromosome inactivation, females can be affected as much as males (3). Plasma α-galactosidase A activity can be determined close to normal values in heterozygous individuals. A definitive diagnosis should be made by molecular genetic analysis in female patients. Only measuring plasma α-galactosidase A activity in female patients may lead to a missed diagnosis. Therefore, as stated in our study, GLA gene sequence analyses were performed in all patients.

Cardiac symptoms and signs may be delayed in female patients with Fabry disease compared with that in male patients. In a study by Linhart et al., pacemaker implantation, myocardial infarction, left ventricular hypertrophy, and conduction defects were observed 8 to 10 years later in female patients diagnosed with Fabry disease (4). According to the Fabry Outcome Survey study data, the mean time between symptom onset and diagnosis was 13.7 and 16.3 years for males and females, respectively (5). left ventricular hypertrophy, considered a high-risk factor for Fabry disease, was observed in 46% males and 28% females in Fabry Outcome Survey. The age at onset of left ventricular hypertrophy in males and females was 38.0 and 55.4 years, respectively (5). According to the Fabry Outcome Survey database, female patients are usually (often severely) affected.

Female patients with chronic kidney disease, left ventricular hypertrophy, and/or stroke, all considered high-risk factors for Fabry disease, should be diagnosed using molecular genetic analyses. However, screening for Fabry disease in our country remains suboptimal. In the future, a countrywide approach to identify Fabry disease among high-risk patients seems warranted.
